# Heat-Responsive PLA/PU/MXene Shape Memory Polymer Blend Nanocomposite: Mechanical, Thermal, and Shape Memory Properties

**DOI:** 10.3390/polym17030338

**Published:** 2025-01-26

**Authors:** Rajita Sanaka, Santosh Kumar Sahu, P. S. Rama Sreekanth, Jayant Giri, Faruq Mohammad, Hamad A. Al-Lohedan, Mohd Shahneel Saharudin, Quanjin Ma

**Affiliations:** 1 School of Mechanical Engineering, VIT-AP University, Besides A.P. Secretariat, Amaravati 522237, Andhra Pradesh, India; 2 Department of Mechanical Engineering, Yeshwantrao Chavan College of Engineering, Nagpur 441110, Maharashtra, India; 3 Division of Research and Development, Lovely Professional University, Phagwara 144411, Punjab, India; 4 Centre for Research Impact & Outcome, Chitkara University Institute of Engineering and Technology, Chitkara University, Rajpura 140401, Punjab, India; 5 Surfactants Research Chair, Department of Chemistry, College of Science, King Saud University, P.O. Box 2455, Riyadh 11451, Saudi Arabia; 6 School of Computing & Engineering Technology, Robert Gordon University, Garthdee Road, Aberdeen AB10 7QB, UK; 7 School of System Design and Intelligent Manufacturing, Southern University of Science and Technology, Shenzhen 518055, China

**Keywords:** PU, MXene, PLA, blend composite

## Abstract

This study investigates the fabrication and characterization of heat-responsive PLA/PU/MXene shape memory polymer blend nanocomposites with varying PLA content (10, 20, 30, and 50%) and a fixed MXene content of 0.5 wt.%. The results indicate significant improvements in mechanical properties, with the 50% PLA/PU/MXene blend showing a 300% increase in ultimate tensile strength and a 90% decrease in % elongation compared to pure PU. Additionally, the 50% blend exhibited a 400% increase in flexural strength. Microstructural analysis revealed dispersed pores and sea–island morphology in pure PU and the 50% PLA/PU/MXene blend. Thermal analysis using DSC showed an increase in crystallinity from 33% (pure PU) to 45% for the 50% PLA/PU/MXene blend, indicating enhanced crystalline domains due to the semi-crystalline nature of PLA and MXene’s influence on molecular ordering. TGA demonstrated a significant improvement in thermal stability, with the onset temperature rising from 185 °C (pure PU) to 212 °C and the degradation temperature increasing from 370 °C to 425 °C for the 50% blend, attributed to the rigid structure of PLA and MXene’s stabilizing effect. Shape memory testing revealed that the 30% PLA/PU/MXene blend achieved the best shape fixity and recovery with optimal performance, whereas higher PLA content diminished shape memory behavior.

## 1. Introduction

Polymer blends are a class of advanced materials produced by combining two or more polymers, which shows enhanced properties compared to the individual polymer components [1,2,3]. Polymer blends demonstrate superior mechanical strength, thermal stability, and flexibility. This approach demonstrates a process to develop tailored materials for specific applications without necessitating the synthesis of entirely new polymers [4,5]. This reduces the complexity, time, and cost of fabrication of these advanced materials. As a cost-effective method, polymer blends have attracted widespread attention for engineering versatile materials across various industries. Polyurethane (PU) is a novel polymeric material with shape memory properties based on stimulus responsiveness [6,7]. However, the widespread use of virgin PU is limited due to its high cost and inferior mechanical and thermal properties [8]. Therefore, blending PU with other conventional polymers offers the best alternative [9]. When nanofillers are added to these blends, the resulting materials gain superior properties [10,11,12]. The following section reviews the literature on shape memory PU blends and composites.

Mukti Tyagi et al. [13] investigated the mechanical and shape memory properties of PC/TPU blends at various levels of TPU content. It was noted that increased TPU concentration lead to a reduction in tensile strength and an enhancement in elongation, and a 40% TPU blend led to peak performance, displaying superior tensile strength and excellent shape memory properties. Lin et al. [14] used the melt-compounding method to fabricate PP/TPU blends and investigated the crystallization attributes, morphological characteristics, and impact properties. Their research revealed that the PP90/TPU10 blend exhibited superior tensile strength and thermal stability among all the blends. Bajsic et al. [15] asserted that PP/TPU blends with an 80 wt.% TPU and 20 wt.% PP composition failed to achieve the desired mechanical attributes, owing to immiscibility and incompatibility. Kim et al. [16] reported on the mechanical behavior of TPU/p-PVC blends. Their findings showed that p-PVC/TPU70 blends exhibited superior mechanical properties owing to miscibility compared to p-PVC/TPU90 blends. Yue et al. [17] investigated the mechanical and structural properties of PVDF/TPU blends. It was noted that, with a 50/50 ratio, mechanical strength is at its lowest due to poor miscibility. However, blends with PVDF concentrations exceeding 50% showed improvements in mechanical strength. Bernardes et al. [18] studied the effect of compatibilization agents, i.e., EBG, on the mechanical behavior of PLA/TPU blends. It was observed that adding EBG improved impact strength by 38% and yield stress by 33%. Raja et al. [19] explored the mechanical and thermal behavior of the PLA/PU/MWCNT blend nanocomposite. The blend was fabricated at 90:10 (PU/PLA) and filled with 2 to 10 wt.% MWCNT. Compared to pure blend, an improvement in tensile strength and glass transition temperature was noted with CNT addition. McLellan et al. [20] demonstrate that adding 0.5 wt.% MXene to TPU/PLA blends significantly enhances mechanical and thermal properties, while 2 wt.% MXene achieves ∼98% shape recovery in under 14 s. Syahir et al. [21] reported on a mechanical and thermal study of PU/neoprene (CR)/graphene (Gr) blend nanocomposite with variations in CR and GR concentration. The PU/CR/Gr 1 sample showed the best properties, improved tensile strength, and glass transition temperature.

The literature review summarized that combining polyurethane-based shape memory polymers (SMPs) with other polymers, such as conventional thermoplastics and thermosetting materials, can yield enhanced shape memory characteristics alongside improved mechanical and thermal properties. The selection of blending components and their compatibility has a significant role in determining the overall performance of the composite material. The selection of PLA for the blend is due to its biodegradability, mechanical strength, and thermal stability, which complement polyurethane’s flexibility and shape memory properties [22]. Additionally, incorporating nanoparticles into polymer blends has been shown to enhance mechanical properties further. However, there is a notable gap in the research focusing on PLA/PU blends reinforced with MXene fillers using the injection molding technique, which forms the basis of this study’s novelty. The choice of MXene filler is owing to its unique two-dimensional structure, high surface area, and exceptional mechanical properties. MXene’s ability to form strong interfacial interactions with polymer matrices can enhance composites’ mechanical and thermal properties [23]. The primary objective of this study is to fabricate a PLA/PU blend with varying PLA ratios while maintaining a constant 0.5 wt.% concentration of MXene filler. Additionally, the work aims to evaluate the blend’s composite mechanical, thermal, and shape memory properties. The rationale for selecting MXene filler and maintaining a 0.5 wt.% concentration has been detailed in our earlier publication, by Sanaka et al. [24].

## 2. Materials and Methods

### 2.1. Materials

Polyurethane (PU) in pallet dimensions with a size of 6.5–8 mm, T_g_ at 55 °C, and the density was 0.83 g/cm^3^, were sourced from SMP Technologies, based in, Tokyo, Japan. The granular form PLA (polylactic acid) with a size between 6 and 8 mm, a glass transition temperature (T_g_) of 65 °C, and a density between 1.20 and 1.30 g/cm^3^ were sourced from Banka BioLoo Limited, Secunderabad, Telangana, India. MXene (Ti_3_C_2_) nanofiller of size 40–50 nm presented as flaky morphology, with a purity of more than 99% and a density of 3.8 g/cm^3^, and were supplied by Nano Research Elements, India.

### 2.2. Blend Nanocomposite Samples Fabrication

PU and PLA pellets were mixed to prepare various blend compositions based on different weight percentages, namely 10%, 20%, 30%, and 50%, and at a constant 0.5 wt.% concentration of MXene nanofiller. The PLA percentages (10%, 20%, 30%, and 50%) were selected to study the impact of varying levels of thermoplastic content on the blend’s mechanical, thermal, and shape memory properties. The literature indicates that, beyond 50% PLA, compatibility issues with polyurethane can arise, affecting performance [25,26]. The rationale for selecting 0.5 wt.% MXene concentration was allied with the literature [24], which identified it as optimal wt.% for enhancing mechanical and thermal properties without compromising shape recovery, avoiding issues like filler agglomeration at higher concentrations. At first, the 0.5 wt.% chemically modified MXene fillers were sonicated in a sonicator with ethanol (10:1) in a beaker, and then the nanofluid was mixed with the required proportion of PU/PLA blend, kept on a hot plate, and stirred well. Subsequently, these samples underwent dehumidification in a dryer maintained at 80 °C for five hours, before being fed into an injection molding apparatus. Ultimately, the resultant samples were extracted as per ASTM standards from the die of the injection molding machine. The prepared samples were pure PU, 10% PLA/PU/MXene blend, 20% PLA/PU/MXene blend, 30% PLA/PU/MXene blend, and 50% PLA/PU/MXene blend.

### 2.3. Microstructure

A scanning electron microscopy (SEM) ZEISS EVO10, Jena, Germany was utilized to analyze the microstructure of the samples.

### 2.4. Mechanical Test

Tensile testing was executed utilizing the Tinius Olsen H10 KL UTM apparatus. The experiments were performed following ASTM D638 (Type-V) standards. The test was executed at ambient temperature, at a 2 mm/min strain rate for each test. Five iterations of testing on the same test specimen were performed, with the mean result documented. Flexural tests were carried out on the UTM equipment, assisted by additional provisions (Tinius Olsen H10KL equipment), with an 80 mm gauge length along the standards of ASTM D790, the dimensions of which were 125 × 15 × 5 mm^3^, and are given due consideration. The strain rate was 1 mm/min, and the test was executed at room temperature. Five repetitions of testing were performed, and the average outcomes were recorded.

### 2.5. Thermal Properties

Differential scanning calorimetry (DSC) and thermogravimetric analysis (TGA) tests were conducted on the SAT 8000 model from PerkinElmer, Waltham, MA, USA, having a temperature operating range of 25–600 °C and a constant heating rate, as well as cooling rate, of 20 °C/min, respectively. The sample mass for the analysis was approximately 25 mg. The nitrogen gas testing environment was maintained with a 20 mL/min flow rate. Five test repetitions for each sample were carried out to confirm repeatable results.

### 2.6. Shape Memory Test

The shape memory test was executed using fold–deploy methodology on specimens with dimensions of an 80 × 10 × 2 mm^3^ rectangular sample. The test consisted of several phases. Firstly, the samples were exposed to elevated temperatures at 80 °C (>Tg), utilizing a heating apparatus for 5 min. Subsequently, the specimen was bent into a “U” shape configuration and temporarily secured. Then, the sample was swiftly immersed in water maintained at ambient temperature. The experimental procedure was conducted over three discrete cycles for all the samples. Equations (1) and (2) were used to evaluate the safe fixity ratio (R_f_) and the shape recovery ratio (R_r_) [27]:

(1) $$R_{f}=\frac{\left(\theta_{i}-\theta_{f}\right)}{\theta_{i}}\times 100\%$$

(2) $$R_{r}=\frac{\theta_{f}}{180{}^{\circ}}\times 100\%$$

The notation here is $\theta_{i}$: initial angle, and $\theta_{f}$: final angle.

## 3. Results and Discussion

### 3.1. Tensile Properties

**Figure 1:**
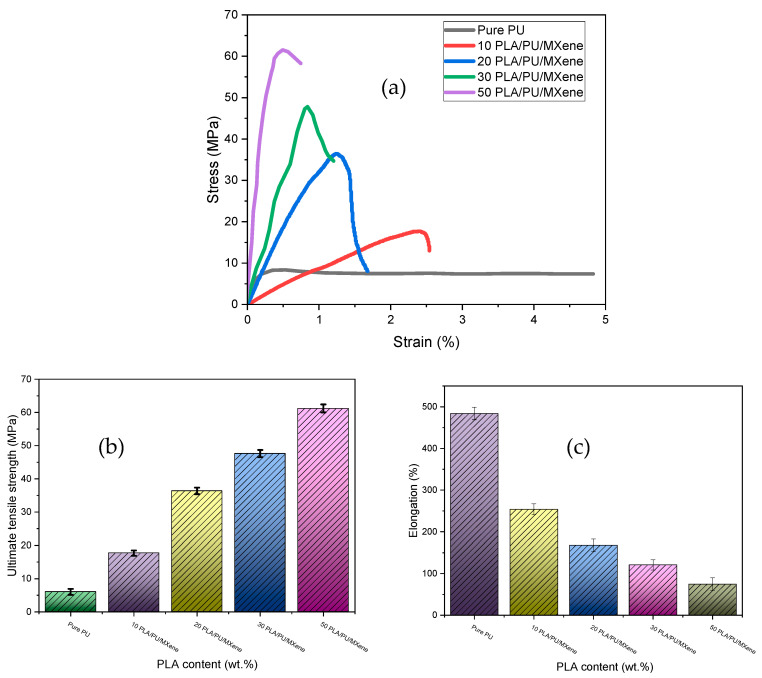
(**a**) Stress vs. strain, (**b**) ultimate tensile strength, (**c**) % elongation for pure PU, 10, 20, 30, and 50% PLA/PU/MXene sample.

**Figure 2:**
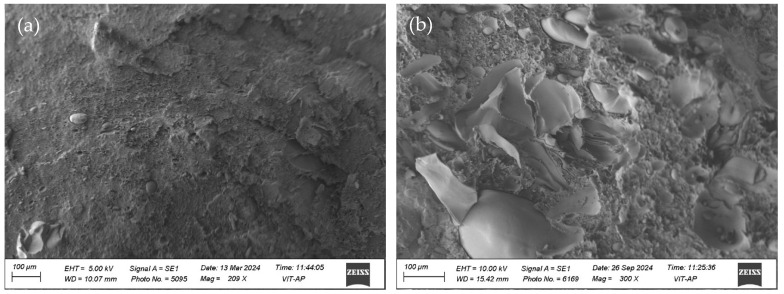
Microstructure of (**a**) pure PU, (**b**) 50 PLA/PU/MXene blend.

Figure 1a represents the stress vs. strain results for all the samples, i.e., pure PU, 10% PLA/PU/MXene blend, 20% PLA/PU/MXene blend, 30% PLA/PU/MXene blend, and 50% PLA/PU/MXene blend. The *x*-axis is strain %, and the *y*-axis shows the stress in MPa. The steepest ascent is demonstrated by a 50% PLA/PU/MXene blend followed by 30, 20, and 10% PLA/PU/MXene blend and a pure PU sample. Figure 1b shows the ultimate tensile strength for all the samples measured from the stress–strain curve. The lowest value of ultimate tensile strength is noted for pure PU, which is improved by 75, 150, 212, and 300% for 10, 20, 30, and 50% PLA/PU/MXene blend, respectively. The % elongation is shown in Figure 1c, which decreases by 48, 69, 79, and 90% for 10, 20, 30, and 50% PLA/PU/MXene blend compared to pure PU, respectively. This suggests that adding more PLA to the PU blend reduces flexibility and increases stiffness. The rise in ultimate tensile strength and decrease in % elongation with blend % (at constant MXene wt.%) is verified from the microstructural analysis. Figure 2a illustrates the microstructure of pure PU, and Figure 2b shows for 50% PLA/PU/MXene blend. Dispersed pores are observed in the pure PU sample, whereas the 50% PLA/PU/MXene blend exhibits a sea–island morphology [28]. Blending PLA and MXene contributes to forming sea–island structures, which partially fill the pore spaces. This decrease in porosity reduces the % elongation results of the composite material and improves its load-bearing capacity, increasing the ultimate strength.

### 3.2. Flexural Properties

**Figure 3:**
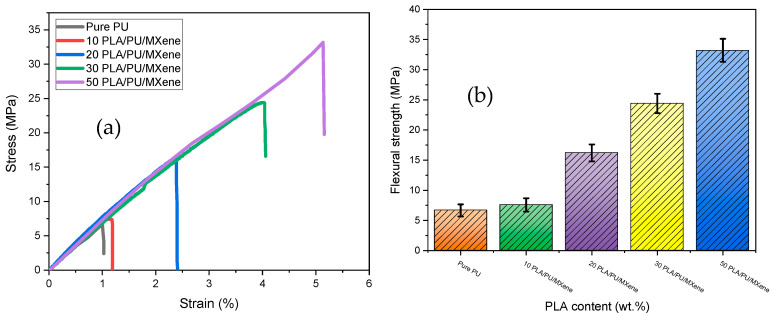
(**a**) Stress vs. strain, (**b**) flexural strength for pure PU, 10, 20, 30, and 50% PLA/PU/MXene sample.

Figure 3a,b show the flexural (three-point bending) test results for pure PU and the PU/PLA/MXene blend at various PLA contents (10, 20, 30, and 50%) and at fixed 0.5 wt.% of MXene. Figure 3a shows the stress vs. strain results where the steepest rise is observed for 50% PLA/PU/MXene. The flexural strength is evaluated from the above graph and shown in Figure 3b. The flexural strength of pure PU, measured at 6.67 MPa, demonstrates significant enhancement with the incorporation of PLA and fixed MXene content. The flexural strength increases by 15%, 142%, 263%, and 400% for the 10%, 20%, 30%, and 50% PLA/PU/MXene blends, respectively. This substantial improvement is attributed to the increased stiffness and reduced ductility introduced by the rigid PLA matrix, coupled with the reinforcing effect of MXene [29].

### 3.3. Thermal Behavior

#### 3.3.1. DSC

**Figure 4:**
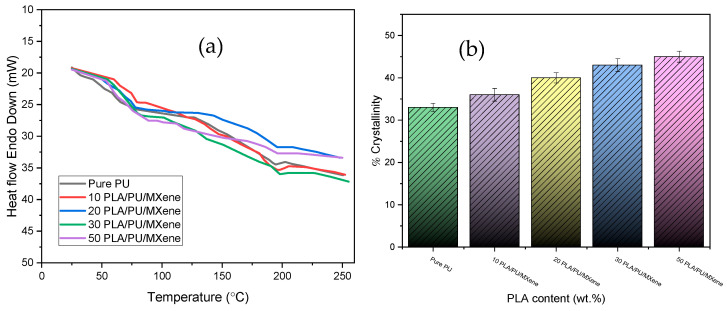
(**a**) Heat flow vs. temperature, (**b**) % crystallinity for pure PU, 10, 20, 30, and 50% PLA/PU/MXene sample.

Pure polyurethane (PU) and blended, i.e., 10, 20, 30, and 50% PLA/PU/MXene blends, composites samples were examined using differential scanning calorimetry (DSC) to explore the thermograms, i.e., glass transition (T_g_), melting temperature (T_m_), and crystallinity features, i.e., % crystallinity. The graph in Figure 4a shows the relationship between heat flow and temperature, which shows an increase in temperature with the heat flow for all samples. Table 1 outlines PU’s DSC results and PLA/PU/MXene blend composites. It is observed that the glass transition temperature (T_g_) for pure PU occurs at 55 °C and shifts to 56 °C, 57 °C, 60 °C, and 64 °C for the 10, 20, 30, and 50% PLA/PU/MXene blends, respectively. For the pure PU, the melting temperature (T_m_) is 193 °C and increases linearly to 195 °C, 196 °C, 197 °C, and 198 °C for the 10, 20, 30, and 50% PLA/PU/MXene blends, respectively. Figure 4b shows the % crystallinity results for all the samples evaluated using Equation (3) [30].

(3) $$X_{c}=\frac{∆H_{m}}{∆H_{m}^{0}(1-N)}\times 100\%$$

where $∆H_{m}$ and $∆H_{m}^{0}$ are the melting enthalpies (J/g) of the blend composite and 100% crystalline PLA sample, respectively, and N is the PU fraction in the blend.

It is witnessed that pure PU % crystallinity is 33%, which is augmented to 36, 40, 43, and 45% C for the 10, 20, 30, and 50% PLA/PU/MXene blends, respectively. The increased melting temperature (T_m_) and % crystallinity for PLA/PU/MXene blend composites are attributed to the restricted molecular mobility and stronger intermolecular interactions caused by the addition of PLA and MXene. PLA, a semi-crystalline polymer, introduces rigid crystalline domains into the blend, requiring higher thermal energy to melt and thereby increasing the overall crystallinity. Moreover, the inclusion of MXene, a 2D nanomaterial, improves the dispersion of PLA and establishes strong interfacial bonding between the polymer chains and the filler, further enhancing the composite’s structural properties [31].

#### 3.3.2. TGA and DTG

**Figure 5:**
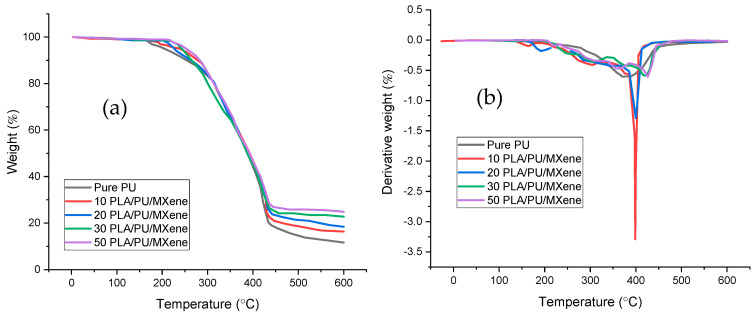
(**a**) Weight %, (**b**) derivative weight % vs. temperature for pure PU, 10, 20, 30 and 50% PLA/PU/MXene sample.

The TGA results examine the thermal degradation behavior of pure PU and PLA/PU/MXene blend composites (i.e., 10, 20, 30, and 50%). Figure 5a shows the TGA curve for all the samples, and the samples show gradual weight loss with an increasing temperature, indicating thermal decomposition. The highest onset temperature, corresponding to approximately 5% weight loss, is observed for the 50% PLA/PU/MXene blend, at 212 °C. This value decreases to 210 °C, 204 °C, 190 °C, and 185 °C for the 30, 20, 10% PLA/PU/MXene blends and pure PU, respectively. This implies that the onset temperature is proportional to the increase in the PLA content, which yields an increase in thermal stability by incorporating PLA and MXene [32]. Figure 5b presents the differential thermogravimetric (DTG) curves for all samples. The DTG curve, derived from the TGA data, helps identify the degradation temperature corresponding to approximately 50% weight loss. The highest degradation temperature is noted for the 50% PLA/PU/MXene blend composite sample, i.e., 425 °C, which decreased to 419, 401, 398, and 370 °C for 30, 20, 10% PU/PLA/MXene blend, and pure PU, respectively. The higher degradation temperature observed in the 50% PLA/PU/MXene composite is primarily due to the increased PLA content. PLA’s superior thermal stability, attributed to its higher degradation temperature, significantly enhances the composite’s thermal resistance as its content increases. Its rigid structure forms a stable network, reducing decomposition rates. Additionally, MXene’s uniform interaction with both polymers creates a synergistic effect, further maximizing the thermal stability of the blend [33].

### 3.4. Shape Memory Behavior

**Figure 6:**
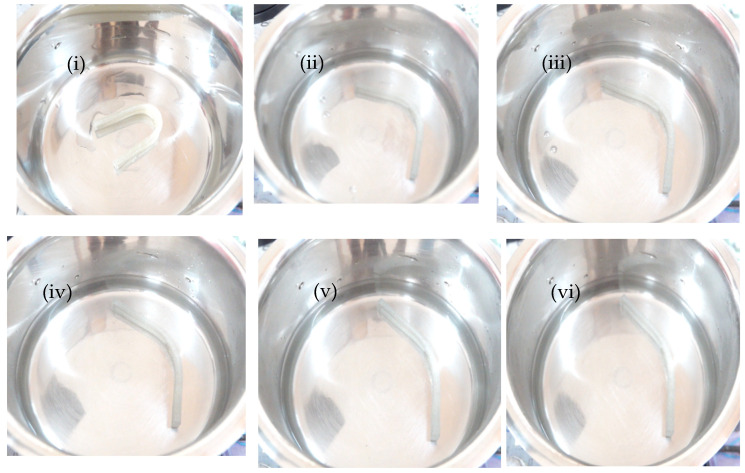
Pure PU at (**i**) 10 s, (**ii**) 20 s, (**iii**) 30 s, (**iv**) 40 s, (**v**) 50 s, and (**vi**) 60 s in the cycle 1 shape memory test [24].

**Figure 7:**
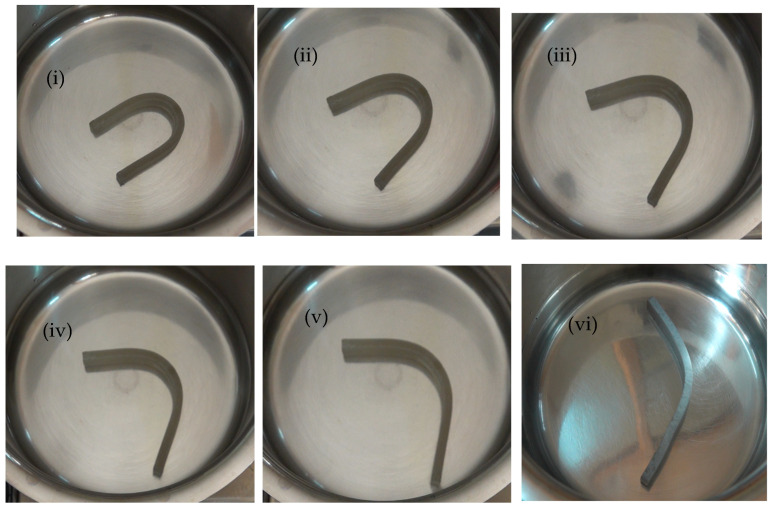
30 PLA/PU/MXene at (**i**) 10 s, (**ii**) 20 s, (**iii**) 30 s, (**iv**) 40 s, (**v**) 50 s, and (**vi**) 60 s in the cycle 1 shape memory test.

**Figure 8:**
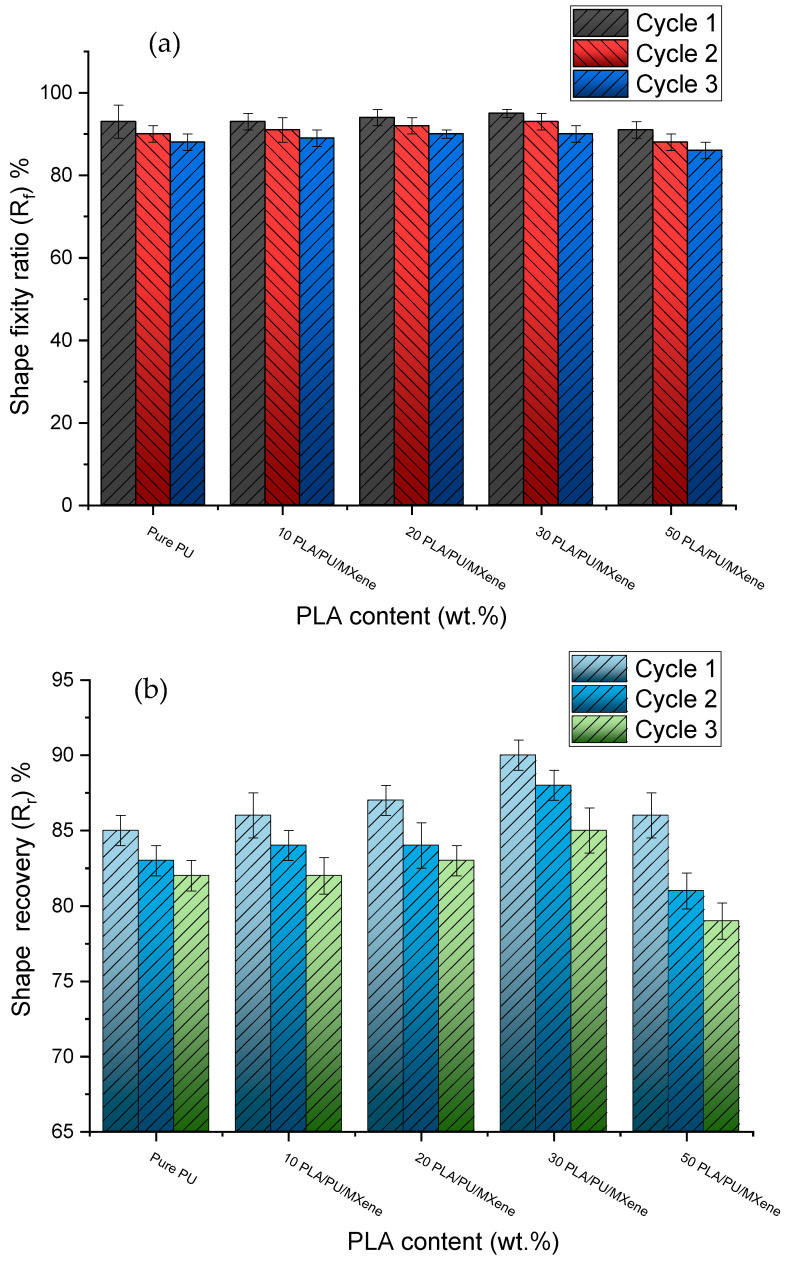
(**a**) Shape fixity (R_f_), (**b**) shape recovery (R_r_) ratio for pure PU, and the 10, 20, 30 and 50% PLA/PU/MXene sample.

Figure 6 and Figure 7 illustrate the deformation states observed during the shape memory test for the first cycle, conducted as per Section 2.6, for pure PU and the 30% PU/PLA/MXene blend nanocomposite samples. The test was repeated for three cycles, and the results were recorded. As shown in Figure 8a, during cycle 1, the shape fixity ratio (R_f_) of pure PU is approximately 93%. For the 10% PU/PLA/MXene blend, the R_f_ remains unchanged at 93%, similar to pure PU. However, with 20% and 30% PU/PLA/MXene blends, R_f_ increases by 1.08% (94.08%) and 2.15% (95.15%), respectively. Conversely, for the 50% PU/PLA/MXene blend, a decrease of 2.15% (90.85%) in R_f_ is observed. A similar trend is observed at cycle 2 and 3. These results indicate that the addition of up to 30% PLA/PU/MXene enhances the R_f_, whereas excessive PLA content (50%) adversely affects shape fixity. The improvement in R_f_ at 10–30% is attributed to the balanced interplay between the flexibility of PU and the rigidity of PLA. The addition of PLA increases crystallinity, rigidity, and interfacial interactions, which collectively enhance shape retention [34].

Figure 8b presents the shape recovery ratio (R_r_) for all samples. During cycle 1, the R_r_ of pure PU is approximately 85%, which increases by 1.18% (86.18%), 2.35% (87.35%), and 5.88% (90.88%) for 10%, 20%, and 30% PU/PLA/MXene blends, respectively. For the 50% blend, R_r_ increases marginally by 1.17% (86.17%) compared to pure PU. In cycle 2, the R_r_ of pure PU decreases to 83%, while the 10%, 20%, and 30% blends show improvements of 1.2% (84.2%), 1.2% (84.2%), and 6.02% (89.02%), respectively. However, for the 50% blend, a reduction of −2.41% (80.59%) in R_r_ is noted. Similarly, in cycle 3, the R_r_ of pure PU further decreases to 82%, with no change observed for the 10% blend. The 20% and 30% blends; however, exhibit increases of 1.22% (83.22%) and 3.66% (85.66%), respectively, while the 50% blend demonstrates a decrease of −3.66% (78.34%) in R_r_. The improved R_r_ for 30% blends is due to the synergistic interaction between the flexibility of PU and the rigidity and crystallinity introduced by PLA, resulting in superior shape recovery properties. Beyond 30%, the increased PLA content leads to diminished R_r_, primarily due to reduced elasticity, excessive crystallinity, and loss of flexibility, which negatively affect the recovery ability [35].

## 4. Conclusions

This study demonstrates the fabrication and characterization of heat-responsive PLA/PU/MXene shape memory polymer blend nanocomposite at various blend % PLA (i.e., 10, 20, 30, and 50%) in PU and fixed MXene content of 0.5 wt.%. The following conclusions are drawn from the research:

- The 50% PLA/PU/MXene blend nanocomposite showed a 300% upsurge in ultimate tensile strength and a 90% decrease in % elongation compared to pure PU.
- The highest flexural strength value is observed for the 50% PLA/PU/MXene blend nanocomposite sample, which increased to 400% from pure PU.
- The microstructure results confirmed the dispersed pores and sea–island morphology for pure PU and 50% PLA/PU/MXene blend nanocomposite sample.
- The DSC results show an increase in % crystallinity from 33% (pure PU) to 45% for the 50% PLA/PU/MXene blend nanocomposite, highlighting enhanced crystalline domains due to PLA’s semi-crystalline nature and MXene’s role in improving molecular ordering and interfacial bonding.
- The onset temperature increased from 185 °C (pure PU) to 212 °C (50% PLA/PU/MXene), while the degradation temperature rose from 370 °C to 425 °C, highlighting improved thermal stability due to PLA’s rigidity and MXene’s stabilizing effect.
- The 30% PLA/PU/MXene blend exhibited the best shape fixity and recovery ratios across cycles, achieving an optimal balance between PLA’s rigidity and PU’s flexibility, while excessive PLA content reduced performance.

The study is limited to a fixed 0.5 wt.% MXene and does not explore higher or lower MXene concentrations. Future research could investigate the effects of varying MXene contents, employ 4D printing, and include tests like tribology, DMA, and rheology to enhance the performance and expand its applications in advanced fields such as soft robotic hands [36,37,38], thermo-responsive sensors [39,40,41], endovascular applications [42,43,44], and artificial muscle material [45,46,47].

## Figures and Tables

**Table 1:** DSC results of various samples.

Sample	Glass Transition Temp (T_g_) (°C)	Melting Temp (T_m_) (°C)	Enthalpy of Melting (ΔH_m_)(J/g)	% Crystallinity
Pure PU [24]	55	193	-	33
10% PLA/PU/MXene	56	195	3	36
20% PLA/PU/MXene	57	196	7	40
30% PLA/PU/MXene	60	197	12	43
50% PLA/PU/MXene	64	198	21	45

## Data Availability

The raw data supporting the conclusions of this article will be made available by the authors upon request.

## Abbreviations

4D	Four-dimensional
SMP	Shape memory polymer
SMPC	Shape memory polymer composite
TPU	Thermoplastic polyurethane
PLA	Polylactic acid
PU	Polyurethane
MXene	Titanium carbide (Ti_3_C_2_)
TGA	Thermogravimetric analysis
DSC	Differential scanning calorimetry
DMA	Dynamic mechanical analysis
SEM	Scanning electron microscopy
ASTM	American Society for Testing and Materials
T_g_	Glass transition temperature
T_m_	Melting temperature
R_f_	Shape fixity ratio
R_r_	Shape recovery ratio
CNT	Carbon nanotube
Gr	Graphene
CR	Chloroprene rubber
PC	Polycarbonate
PP	Polypropylene
PVDF	Polyvinylidene fluoride
p-PVC	Plasticized polyvinyl chloride
DTG	Derivative thermogravimetric analysis
SMPU	Shape memory polyurethane
wt.%	Weight percentage
EBG	Ethylene butyl acrylate copolymer
